# Real-world outcomes of TMVR-eligible and TMVR-ineligible patients

**DOI:** 10.1093/ehjimp/qyaf098

**Published:** 2025-08-06

**Authors:** Edoardo Zancanaro, Nicola Buzzatti, Nicolò Azzola Guicciardi, Paolo Denti, Eustachio Agricola, Francesco Ancona, Ottavio Alfieri, Michele De Bonis, Francesco Maisano, Roberto Lorusso

**Affiliations:** Department of Cardiac Surgery, San Raffaele Scientific Institute, Via olgettina 69, Milano 20140, Italy; Cardiovascular Imaging Unit, San Raffaele Scientific Institute, Via olgettina 69, Milano 20140, Italy; Cardiovascular Research Institute (CARIM), University of Maastricht, UNS 50, Room H1, Universiteitssingel 50, 632, 6229 ER Maastricht, The Netherlands; Department of Cardiac Surgery, San Raffaele Scientific Institute, Via olgettina 69, Milano 20140, Italy; Department of Cardiac Surgery, San Raffaele Scientific Institute, Via olgettina 69, Milano 20140, Italy; Department of Cardiac Surgery, San Raffaele Scientific Institute, Via olgettina 69, Milano 20140, Italy; Cardiovascular Imaging Unit, San Raffaele Scientific Institute, Via olgettina 69, Milano 20140, Italy; Cardiovascular Imaging Unit, San Raffaele Scientific Institute, Via olgettina 69, Milano 20140, Italy; Department of Cardiac Surgery, San Raffaele Scientific Institute, Via olgettina 69, Milano 20140, Italy; Department of Cardiac Surgery, San Raffaele Scientific Institute, Via olgettina 69, Milano 20140, Italy; Department of Cardiac Surgery, San Raffaele Scientific Institute, Via olgettina 69, Milano 20140, Italy; Cardiovascular Research Institute (CARIM), University of Maastricht, UNS 50, Room H1, Universiteitssingel 50, 632, 6229 ER Maastricht, The Netherlands; Heart and Vascular Centre, Maastricht University Medical Centre, Maastricht, The Netherlands

**Keywords:** TMVR, TENDYNE, mitral, MR

## Abstract

**Aims:**

Over the past decade, transcatheter valve replacement has emerged as a therapy for selected patients with valvular heart. Clinical experience with transcatheter mitral valve replacement (TMVR) has been limited to date and provides little insight into its potential as a viable therapy for MR. The present study aims to analyze the current longest follow-up real-life outcomes of TMVR procedures with a specific focus on the patient population left untreated due to the unfeasibility of the procedure.

**Results:**

Out of 3400 patients referred for mitral pathology, 88 were screened for TMVR procedure, being unfeasible for surgical and TEER procedure (Transcatheter Edge-to-Edge Repair). 37 pts (45%) were screened positive and treated with TMVR; 30 (81%) with Tendyne system (Abbott) and 7 (19%) with Tiara. For cardiac death, in TMVR the survival was 97.2%, 90.7%, and 90.7% at 1, 2, and 4 years, respectively. Concerning MT, instead, it was 86.4%, 77%, and 42% at 1, 2, and 4 years, respectively. A difference is seen between the two groups, *P*-value 0.024.

**Conclusion:**

TMVR is a valid option in selected patients and give valid longer follow-up results. The TMVR-ineligible patients showed a progressive detrimental worse survival across the follow-up.

## Graphical Abstract

**Graphical Abstract:**
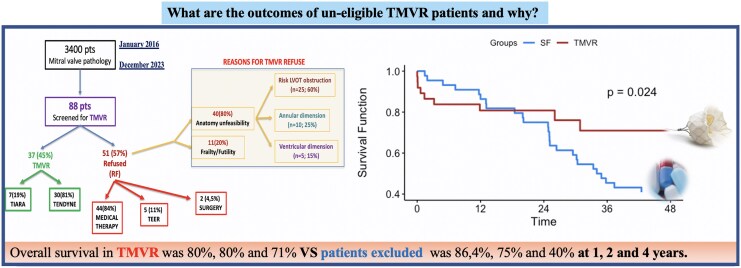

## Introduction

Mitral valve regurgitation (MR) is common, with an estimated prevalence of 2 to 4 million people in the United States alone.^1^ The prevalence is age-dependent, affecting >6% of those aged > 65 years, and is expected to increase with current demographic trends. The prognosis of untreated MR is poor, with progressive left ventricular (LV) dilation, myocardial dysfunction, and cardiac failure, leading to substantial morbidity and mortality, and a considerable economic burden.^2^ Despite current practice guidelines, which advocate surgery for patients with symptoms of LV systolic dysfunction, the majority of patients with severe MR do not undergo surgery.^3^ The reasons include high-surgical-risk from advanced age or multiple comorbidities and a lack of clear data supporting valve surgery for secondary MR with LV dysfunction.^1–3^ Over the past decade, transcatheter valve replacement has emerged as a therapy for selected patients with valvular heart disease,^4,5^ particularly those with severe aortic valve stenosis. Although *trans*-catheter aortic valve replacement has become the standard of care for high-surgical-risk patients with aortic stenosis, the regurgitant mitral valve poses unique challenges for successful transcatheter therapy. Clinical experience with transcatheter mitral valve replacement (TMVR) has been limited to date and provides little insight into its potential as a viable therapy for MR.^6,7^

The present study aims to analyze the current longest follow-up real-life outcomes of TMVR procedures with a specific focus on the patient population left untreated due to the unfeasibility of the procedure.

## Methods

### Study population and data collection

All patients admitted to the Department of Cardiac Surgery at San Raffaele Scientific Institute between January 2016 and December 2023 with mitral pathology was retrospectively selected for the present study. At the time of admission, all patients underwent Heart-Team discussion and prospective in-hospital data collection into a dedicated database for research purposes.

TMVR screening was assessed by the collaboration between the Heart-Team and the industry expert according to clinical, echocardiographic, and Angio-CT (computed Tomography) reconstructions criteria.

Ethics approval for the study was obtained from our institution’s ethics committee.

### Study endpoints

The primary endpoint of the study was to elucidate the real-life screening process for the TMVR procedure and assess the intra- and post-operative outcomes.

The secondary endpoint was to compare the excluded patients with the TMVR-treated patients.

### Echocardiographic assessment

All patients underwent baseline transthoracic and transoesophageal echocardiograms. Transthoracic echocardiogram was then performed after TMVR before discharge and during follow-up visits. MR was graded following current European recommendations.^8^ A multiparametric approach based on the evaluation of quantitative parameters (effective regurgitant orifice area and regurgitant volume) and also semi-quantitative parameters (Vena contracta width and Color-jet Area), was used to assess MR severity. Right ventricle systolic function was evaluated with tricuspid annular plane systolic excursion (TAPSE), measured with M-mode modality from the tricuspid annular longitudinal excursions in apical 4-chamber view, and with systolic (s’) wave of tissue Doppler velocity imaging (TDI), of basal RV free wall from 4-chamber view. Moderate-to-severe RV systolic dysfunction was defined as TDI s’ wave < 8 cm/s and TAPSE < 13 mm. Nevertheless TMVR screening process was based on the use of Angio-CT protocol with model reconstruction to establish the NEO-LVOT area and possible LVOTO (LVOT-Obstructions) as well as the exact position for transapical approach.

### Statistical analysis

Categorical data were described as absolute and percentage (%) frequency values and compared with the χ^2^ or the Fisher exact tests, as appropriate. Continuous variables were expressed median [25th percentile; 75th percentile] and compared with Mann–Whitney test for independent samples. Kaplan-Meier method was used to estimate overall survival and cardiac death.

Cox regression analysis was employed to assess predictors of all cause death and cardiac death. The multivariate analysis was performed for the variables with a *P*-value < 0.1 at the univariate.

A *P* value <0.05 was set as significant. All analyses were performed using R Studio statistical software (version 2023.09.01-494).

## Results

### Patient cohort characteristics

Out of 3400 patients referred for mitral pathology, 88 were screened for TMVR procedure, being unfeasible for surgical and TEER procedure (Transcatheter Edge-to-Edge Repair). 37 pts (45%) were screened positive and treated with TMVR; 30 (81%) with Tendyne system (Abbott) and 7 (19%) with Tiara. 51 patients instead were screened negative for TMVR. 44 (84%) underwent MD (Medical Therapy alone), 5 (11%) TEER, and 2 (4.5%) surgery due to haemodynamic instability. The main reasons for screening failure were in 40 (80%), anatomy unfeasibility, and 11 (20%) for frailty and futility. The main reasons for anatomy unfeasibility were in 25 (60%) patients, the risk of LVOT obstruction, in 10(25%) unfeasible annular dimension for implant (too small or too large), and 5 (15%) unfeasible ventricular dimension. In *Table 1* is reported the flow chart.

**Table 1:** Screening pathway for TMVR and MR

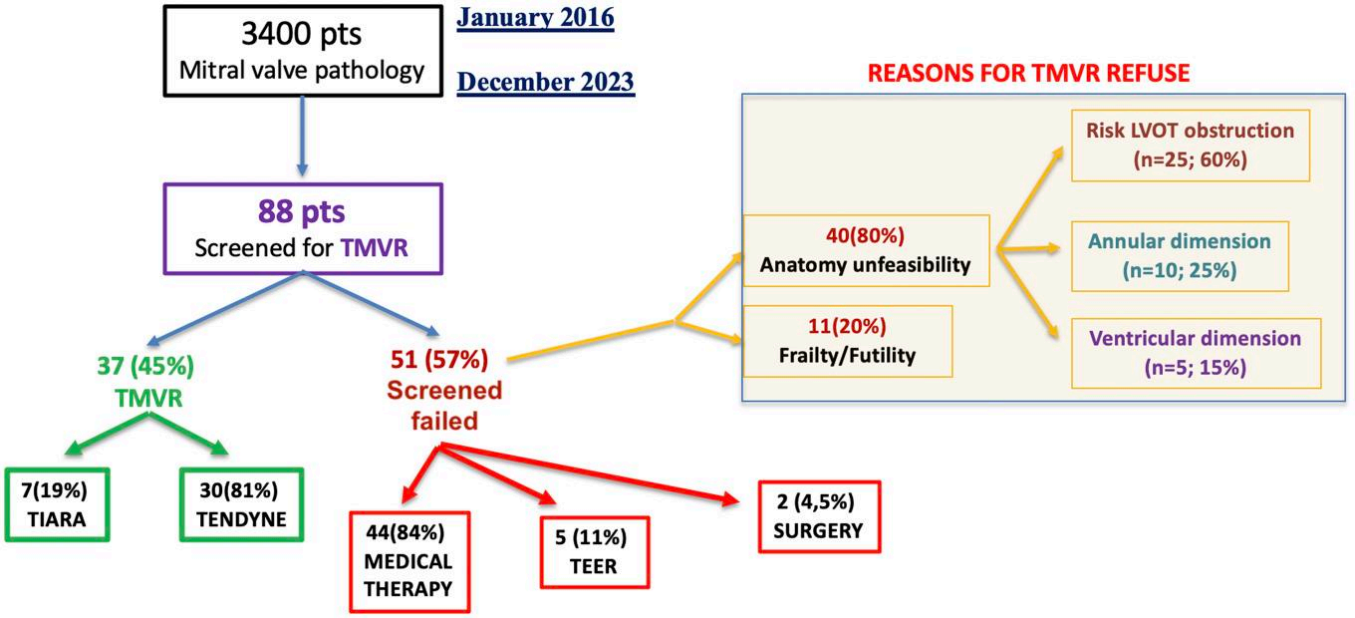

The two groups of study are patients treated with TMVR (TMVR-group), [37 pts (45%)], and patients screened out and treated with optimization of medical therapy alone (MT-group), [44 (84%)].

In *Table 2*, the baseline characteristics of the total cohort as well as the two groups are reported.

**Table 2:** Baseline characteristics of the study population

Baseline characteristics	Overall *n* = 81	MT *n* = 44	TMVR *n* = 37	*P*-value
FEMALE	38 (47%)	17 (39%)	21 (57%)	0.104
BMI	24 (23, 27)	24 (24, 27)	24 (23, 27)	0.827
AGE	79 (75, 84)	82 (78, 86)	75 (71, 79)	0.002
NYHA				0.073
2	16 (20%)	12 (27%)	4 (11%)	
3	53 (65%)	24 (55%)	29 (78%)	
4	12 (15%)	8 (18%)	4 (11%)	
AMI	19 (23%)	6 (14%)	13 (35%)	0.023
SMOKING	17 (21%)	8 (18%)	9 (24%)	0.491
DIABETES	24 (30%)	8 (18%)	16 (43%)	0.014
DISLIPIDIMIA	19 (23%)	4 (9.1%)	15 (41%)	<0.001
HYPERTENSION	62 (77%)	31 (70%)	31 (84%)	0.158
CKD	34 (42%)	13 (30%)	21 (57%)	0.013
CREA	1.19 (1.00, 2.00)	1.00 (1.00, 2.00)	1.82 (1.19, 2.00)	<0.001
EGFR	56 (35, 80)	80 (44, 80)	40 (35, 58)	<0.001
COPD	26 (32%)	5 (11%)	21 (57%)	<0.001
ANGINA	1 (1.2%)	0 (0%)	1 (2.7%)	0.457
STROKE/TIA	6 (7.4%)	4 (9.1%)	2 (5.4%)	0.534
AFIB	28 (35%)	18 (41%)	10 (27%)	<0.001
EXTRA-CARDIAC VASCULOPATIES	16 (20%)	8 (18%)	8 (22%)	0.699
REDO	23 (28%)	10 (23%)	13 (35%)	0.217
PCI	19 (23%)	6 (14%)	13 (35%)	0.023
ICD CRT	11 (14%)	3 (6.8%)	8 (22%)	0.015
EUROSCORE II	13 (8, 20)	13 (8, 18)	16 (9.5, 20)	0.621
STS	20 (12, 23)	19.5 (11.2, 22.7)	20 (18.5, 25)	0.161

CKD, chronic kidney disease; COPD, chronic obstructive pulmonary disease; DM, diabetes mellitus; eGFR, estimated glomerular filtration rate; LVEDD, left ventricle end diastolic diameter; LVEDV, left ventricle end diastolic volume; LVEF, left ventricular ejection fraction; LVESD, left ventricle end systolic diameter; LVESV, left ventricle end systolic volume; MI, myocardial infarction; NYHA, New York Heart Association; S′ TDI, systolic wave of tissue Doppler imaging; sPAP, systolic pulmonary artery pressure; STS, society of thoracic surgeons; TAPSE, tricuspid annular plane systolic excursion; TDI, tissue doppler imaging; TR, tricuspid regurgitation.

TMVR-group showed more clinical sickness with more previous AMI (acute myocardial infarction) (*P*-value 0.023), with more coronary pathology (*P*-value 0.001), worse renal function (creatinine & EGFR, *P*-value 0.001), and worse lung capacity (COPD with *P*-value 0.001). Nevertheless, the MT group had more Atrial fibrillation (*P*-value 0.001) and were older (*P*-value 0.023).

At pre-operative echocardiogram, mitral regurgitation (MR) and mitral stenosis were both present in the total cohort, with a significant majority for MR, *Table 3*. More than 53% of MR aetiology for the total cohort was primary, with a difference between the two groups (*P*-value 0.041). The mean LVEF (left ventricle ejection fraction) was 45% without difference between groups. TMVR-group showed larger LV volume (195 mL/cm) (*P*-value 0.025) and lower sPAP (*P*-value 0.004), *Table 3*.

**Table 3:** Pre-operative echocardiogram (TEE/TTE) parameters

Echo pre-operative	Overall *N* = 81	MT *n* = 44	TMVR *n* = 37	*P*-value
MS				0.043
0	72 (89%)	42 (95%)	30 (81%)	
1	7 (8.6%)	1 (2.3%)	6 (16%)	
4	2 (2.5%)	1 (2.3%)	1 (2.7%)	
MR				0.683
3	6 (7.4%)	4 (9.1%)	2 (5.4%)	
4	75 (93%)	40 (91%)	35 (95%)	
MR AETIOLOGY				0.041
DEGENRATIVE	23 (53%)	17 (68%)	6 (33%)	
FUNCTIONAL	19 (44%)	8 (32%)	11 (61%)	
POST-IRRADIATION	1 (2.3%)	0 (0%)	1 (5.6%)	
AS				0.457
0	80 (99%)	44 (100%)	36 (97%)	
2	1 (1.2%)	0 (0%)	1 (2.7%)	
AR				0.013
0	67 (83%)	41 (93%)	26 (70%)	
2	2 (2.5%)	0 (0%)	2 (5.4%)	
TR				0.092
0	21 (26%)	13 (30%)	8 (22%)	
4	4 (4.9%)	2 (4.5%)	2 (5.4%)	
LVEF	45 (35, 50)	46 (36, 56)	40 (35, 50)	0.098
LVEDV	155 (130, 188)	154 (128, 165)	195 (140, 226)	0.025
LVESV	128 (76, 144)	NA (NA, NA)	128 (76, 144)	
LVEDD	55 (54, 60)	55 (55, 60)	56 (54, 60)	0.413
LEFT ATRIUM DIAMETER	50 (45, 55)	50 (45, 55)	50 (45, 55)	0.859
SPAP	45 (40, 50)	46 (45, 59)	45 (40, 45)	0.004
TDI	10 (9, 11)	10 (9.5, 10)	10.5 (9, 12)	0.105
TAPSE	18.0 (15.0, 20.0)	18.0 (16.0, 18.0)	19.0 (15.0, 20.0)	0.069

### Intra-procedural outcomes

Considering TMVR-group, the intrahospital mortality occurred in 7 patients (19%). 2 surgical conversions were seen, *Table 4*. ECMO was implanted in 3 cases and IABP in 6. Intra-op residual MR = 1+ were seen in 5 pts (16%) while no MR > 1 were documented, *Table 4*.

**Table 4:** Intra-procedural results on the TMVR group

INTRA-operative data	*N* = 37
TIARA	7 (19%)
TENDYNE	30 (81%)
SURGICAL CONVERSION	2 (5.4%)
IABP INTRA-OP	6 (16%)
ECMO INTRA-OP	3 (8.1%)
INOTROPIC SUPPORT	16 (43%)
MR RESIDUAL (intra-op)	
0	27 (84%)
1	5 (16%)

### Post-procedural outcomes

In *Table 5* all the post-operative outcomes and echocardiogram details were reported. Mitral G-mean was 3 mmHg and MR > 1 was still not seen, while residual MR = 1+ was seen in 5 pts (16%). Mean LVEF was 40%. 1 new Pacemaker was implanted, and 7 new A-fib were reported. No stroke was seen. Only 1 patient from MT-group died during the hospitalization due to LCOS (low-cardiac-output-syndrome) and cardiac arrest.

**Table 5:** Post-operative echocardiogram and complications

ECHO + Complication post-OP	*N* = 37
LVEF	40 (30, 50)
MR	
0	27 (84%)
1	5 (16%)
sPAP	35 (35, 40)
TAPSE	19.0 (15.3, 20.0)
RV S TDI	9.75 (11, 12)
MITRAL G-MED	3 (2, 4)
INTRAHOSPITAL DEATH	7 (19%)
NEW PM/ICD	1 (2.7%)
STROKE	0 (0%)
LCOS	7 (19%)
NEW AFIB	7 (19%)
AKI	9 (24%)
IAPB	7 (19%)
ECMO	4 (11%)
IMPELLA	1 (2.7%)
INOTROPIC SUPPORT	28 (76%)
TRASFUSION	21 (57%)
INFECTION	6 (16%)
AMI	0 (0%)

### Follow-up outcomes

Follow-up was 95% complete, with a median follow-up time of 4 years.

**Figure 1:**
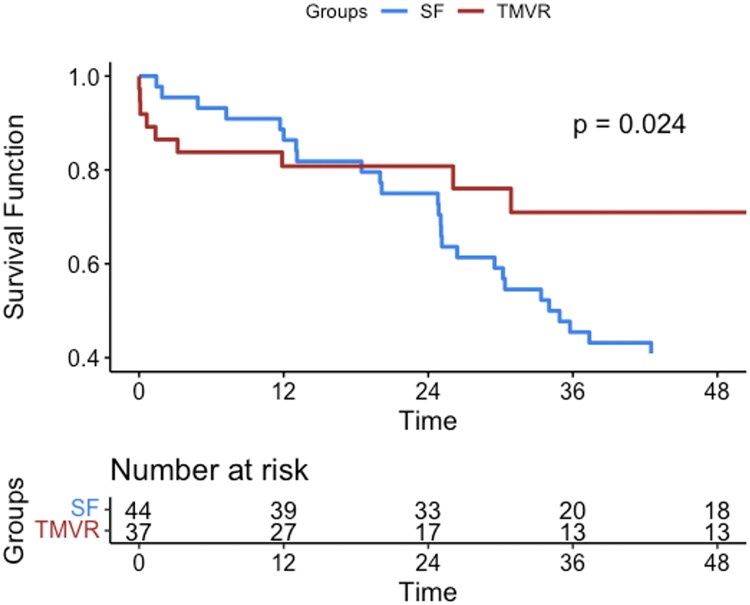
Overall survival in case of TMVR vs. MD at 1, 2, and 4 years.

Overall survival in case of TMVR was 80%, 80%, and 71% at 1, 2, and 4 years, respectively. In case of patients excluded was 86.4%, 75%, and 40% at 1, 2, and 4 years, respectively. Difference is seen at 4 years with a *P*-value 0.024, *Figure 1*.

**Figure 2:**
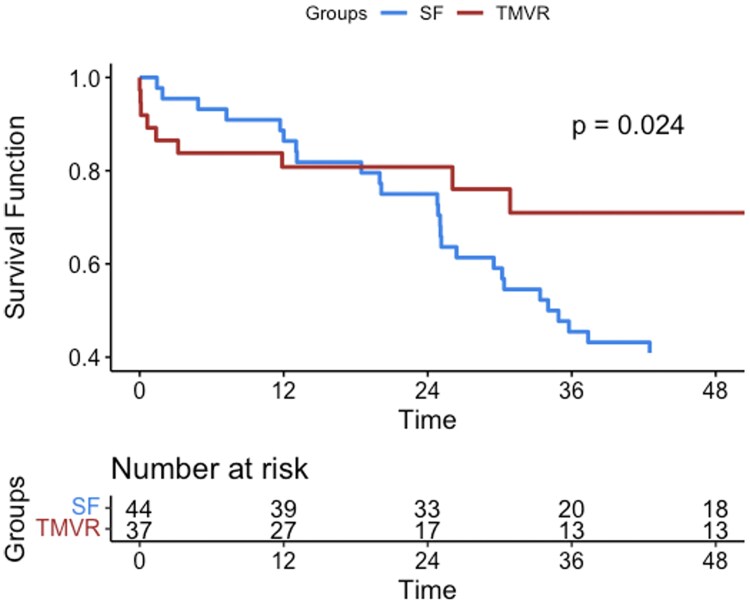
Cardiac death in case of TMVR vs. MD at 1, 2, and 4 years.

For cardiac death, in TMVR the survival was 97.2%, 90.7%, and 90.7% at 1, 2, and 4 years respectively. Concerning MT, instead, was 86.4%, 77%, and 42% at 1, 2, and 4 years respectively. Difference is seen between the two groups, *P*-value 0.024 *Figure 2*.

No NYHA improvement has been recorded in MT patients with 2 cases of HF rehospitalization from the 7 alive at follow-up. No MR/MS improvement has been noted as well.

At univariate analysis, TMVR procedure was protective for overall and cardiac mortality HR 0.45 (IC: 0.22–0.92) (*P*-value = 0.028). At multivariate analysis no variable turned out to be significant, *Table 8*.

**Figure 3:**
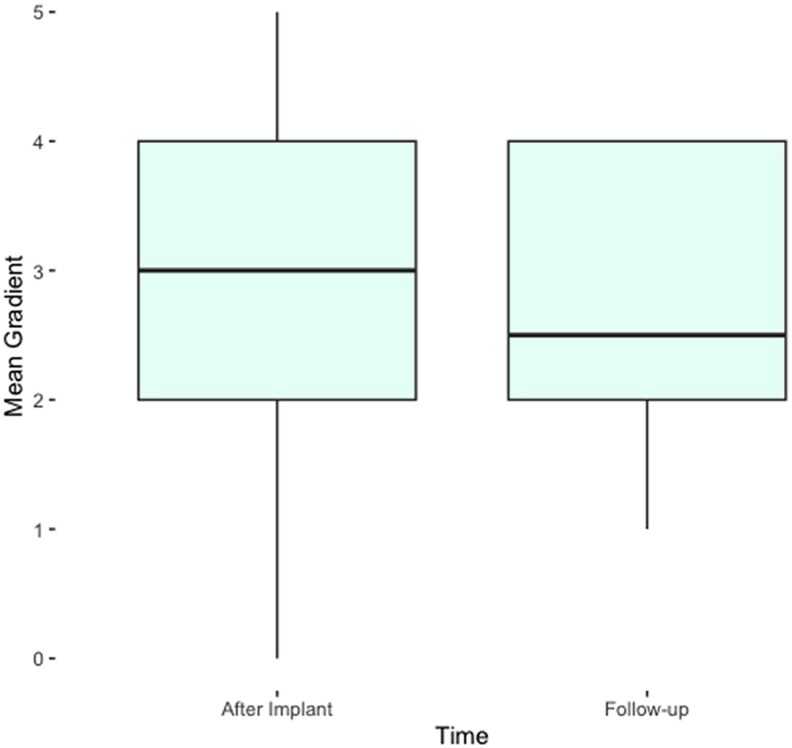
Four years gradient in TMVR group.

TMVR group showed 100% NYHA improvement from the baseline, and solely 2% of the TMVR cohort manifested NYHA class 2 at 4 years, *Table 6*. Only 1 stroke and 1 p.m. implantation are documented at 4 years, *Table 6*. At 4 years, the mean G is 2.5 mmHg and no major complications have been listed. Regarding the mean gradient, it has been noted a reduction over the 4-year follow-up as seen in *Figure 3*. Of note, 1 patient developed the first case of TMVR degeneration and has been treated with a mitral valve-in-valve intervention^9^  *Table 7*.

**Table 6:** Follow-up outcomes and complications

FUP outcomes/complications	*N* = 37
HF HOSPITALIZATION	4 (13%)
CARDIAC SURGERY PROCEDURES	1 (3.3%)
NYHA	
1	25 (93%)
2	2 (7.4%)
NYHA IMPROVEMENT	37 (100%)
NEW PM/ICD	1 (2.7%)
STROKE	1 (2.7%)
CARDIAC ARREST	0 (0%)
NEW AFIB	7 (19%)
AKI	0 (0%)
INFECTIONS	0 (0%)
AMI	0 (0%)
RIVASCOLARIZATION (CABG/PCI)	0 (0%)

**Table 7:** Follow-up echocardiographic data

Echo FUP	*N* = 37
GMED	2.5 (2, 4)
LVEDD	55 (54, 60)
LVEF	46 (35, 50)
sPAP	35 (30, 40)
TAPSE	19.0 (16.8, 20.0)
TR	
2	7 (39%)
3	1 (5.6%)
VALVE THROMBOSIS	0 (0%)
VALVE MIGRATION	0 (0%)
VALVE PVL > MODERATE	0 (0%)
VALVE DYSFUNCTION	1 (2.7%)

**Table 8:** Multivariate analysis on mortality risk

Variable	*P*-value	HR (95% CI)
TMVR screened out	0.892	1.026 (0.711–1.479)
Female	0.847	0.925 (0.417–2.049)
NYHA	0.308	0.128 (0.002–6.680)

## Discussion

This study provides real-world, long-term outcomes following TMVR in patients without surgical or TEER options and compared against patients who screened failed TMVR and were managed medically.

The main findings of the present study are that in a wide patient population referred for treatment of TMVR:

- The majority of the patients were excluded for different reasons (mostly anatomical) and cannot receive TMVR device. They were mostly treated with medical therapy as no other options were available.
- The survival of patients excluded from TMVR treatment is significantly lower compared with those who have been treated, as well as the clinical and echocardiographic state.
- TMVR demonstrated and confirmed the safety not only in the hospital but also at longer follow-up (more than 4 years).

The first important aspect to consider is that, from the total cohort of patients considered for TMVR fewer patients were actually treated than screened out. TMVR has evolving screening access to the present treatment and more patients are becoming eligible for this kind of procedure. Most of the studies performed were regarding selected patients, not regarding the excluded patients and the reasons for unfeasibility. Initially, Muller *et al*.^10^ showed comparable 30-day initial clinical and echocardiographic results.^10^ Also, TMVR has a high in-hospital mortality of 19%.^3^ TMVR yields sustained safety and efficacy outcomes over a 4-year horizon, including low rates of reintervention, stable hemodynamics, and functional improvement despite high rates of peri-procedural mortality.

Interestingly enough, Ludwig *et al*.^11^ showed a ‘tree’ algorithm from their experience to understand the principal reasons of rejections,^11^ as for our experience, most patients were screened out and not treated but the difference note reside in the main reasons; Infact they showed that LV dimension was the principal while for our cohort was the LVOT obstruction (LVOTO) risk. Annular dimension unfeasibility was comparable to our results. The reason for these discrepancies may reside in the different time frame since the present study also analyzed the last generation device and screening process. It is noted that the screening phase for TMVR has become more flexible and it adapted over the years for better compliance in light of the results obtained.

The main lack of Ludwig study was the effectiveness of refusal at long distance and the follow-up in the cohorts analyzed, analyzed instead in the present paper. Yoon *et al*.^12^ showed a similar LVOTO risk as Ludwig in the TMVR screening process and the corresponding poor outcomes in patients treated.^12^ As before, the results reported didn’t take into consideration the new inclusion criteria used.

Once the screening phase is completed, it’s pivotal to discuss the early and late results, with particular attention to patients screened out and left to the medical therapy fate.

The largest cohort study from the CHOICE registry,^13^ TMVR patients showed a slightly worse survival at 1 year with respect to our cohort as well as the patients left in medical therapy. An important consideration to be made is that the cited article compared two different groups of patients; for TMVR, the CHOICE registry was used, and for MT, instead, COAPT patients were used. This may be the main difference with our study, where the patients were selected, excluded or treated in the same institution. Also, the present study is a real-life population where, instead COAPT trial was not very well selected, and the excluded patients were not similar to real-life population, as also shown by Zancanaro *et al*.^14^

Granada *et al*.^13^ did not dispose of a longer follow-up as for the present article; on this aspect it is interesting to notice how the survival rate at 4 years remained stable for TMVR cohort and instead dropped significantly for the refused group, highlighting the detrimental effect of the lack of treatment and the valvular progression pathology if not addressed.

A last word can be said on the lack of statistical difference for survival across groups in Granada^13^ compared with our study; this can be explained by the fact that the cited study analyzed patients from two different trial/registry and the patients used were different and very highly selected; Also no anatomical features were available from the both studies. Nevertheless, it’s also important to mention the lack of matching of the present study but also the real-world selection.

In the clinical (NYHA class status) setting, both studies showed similar results, and this fortifies the importance of treatment on the quality of life of the patients.

Larger numbers and longer follow-up will be needed, however, to validate this finding; nevertheless, the importance of selection is pivotal. On the other hand, the progressive increase in TMVR inclusion will reduce the detrimental effect of the lack of a treatment alternative.

## Limitations

The present was a retrospective single-centre study including a small cohort population; therefore, its findings should be considered hypothesis-generating and will require further data to be confirmed. While assessment of results was carefully performed, retrospective evaluation remains a significant limitation. Finally, the lack of propensity score matching has to be considered, but the real-world screening in a single-centre has not given the possibility for such analysis.

It is essential to acknowledge the lack of propensity-matching in this analysis, which introduces the possibility of confounding by indication. However, this limitation is counterbalanced by the real-world nature of the cohort and the consistency in patient evaluation and management strategies. Unlike multi-registry comparisons, the current study captures a consecutive series of patients assessed under identical conditions, enhancing its internal validity.

Finally, no clear information regarding HF pillars can be provided, imposing a relevant bias in data interpretation.

## Conclusion

TMVR is a valid option in selected patients and is associated with better long-term survival compared with patients who screen fail for TMVR. Despite this, TMVR exclusion rate remains high, and only a small subsets of screened patients have suitable anatomy for device implantation.

## Data Availability

All data are at your disposal for control and check. There is a full disclosure.

## Lead author biography

Edoardo Zancanaro is a cardiac surgery resident that graduated in International Medical Program at Università Vita Salute San Raffaele in Milan, Italy, and he is currently resident at San Raffaele Hospital in Milan. He is also performing an advanced fellowship in Minimally invasive cardiac surgery and Interventional Cardiology at University Hospital in Mainz. The principal goal is to become a Hybrid cardiac surgeon with a specific attention to valvular heart diseases. The major topics of interest are minimally invasive/robotic CS and Transcatheter Valvular Therapy with a close attention to new device/AI incorporated tools to address valvular pathology. He is particular interested into education and the podcast and broadcast that the is leading for EHJIMP and ESC are the main vectors of it.
